# The cerefy® Atlas of Cerebral Vasculature

**Published:** 2010-11-20

**Authors:** Pieter L. Kubben

**Affiliations:** Department of Neurosurgery, Maastricht University Medical Center, The Netherlands

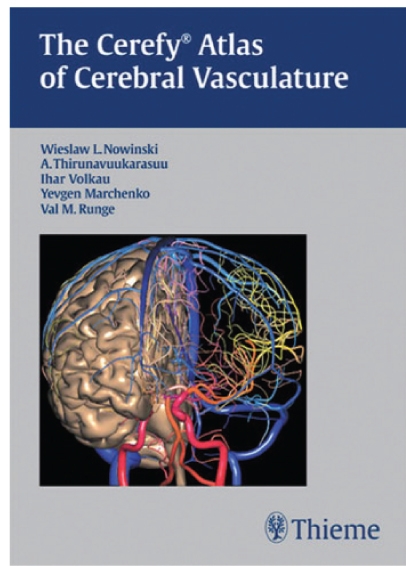



## DESCRIPTION

This interactive CD-ROM offers “an interactive electronic atlas correlating 3D vasculature with surface and sectional anatomy of the human brain” (according to the product’s box). Cerefy is a collaboration between the medical publisher Thieme and the Biomedical Imaging Lab from professor Wieslaw Nowinski in Singapore (funded by the Agency for Science, Technology and Research). More information can be found on http://cerefy.com.

### System requirements

For Windows, the program minimally requires Microsoft Windows XP Service Pack 2 on a 2GHz Intel Core 2 Duo processor, 1 GB RAM and 128 MB video card. For Mac, the minimum requirements are Mac OS X version 10.4 on a 2.8 GHz Intel Core 2 Duo processor, 2 GB RAM and 128 MB video card.

I tested on a 2010-model MacBook Pro i5 2,4 GHz Intel Core 2 Duo with 4GB RAM and NVIDIA GeForce GT 330M 256 MB video card.

## FIRST IMPRESSION

**Figure 1:**
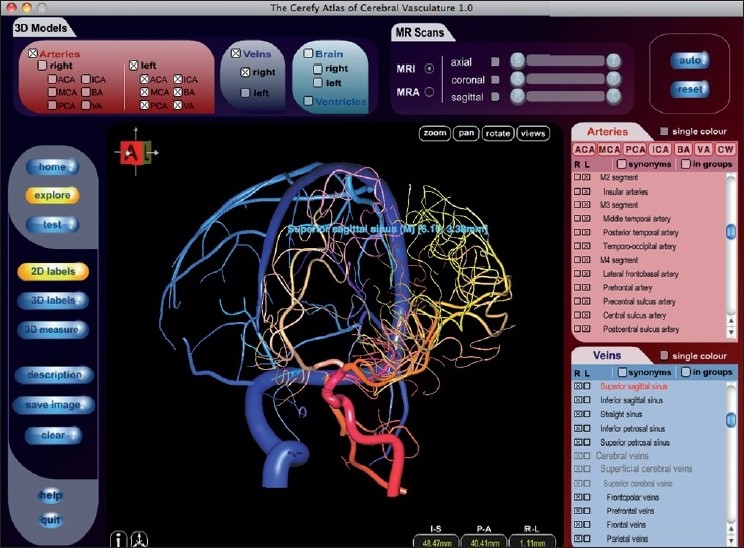
The application’s main screen and navigation structure.

Installation was problem-free, and starting the program was intuitive. While the quality of the images is impressive, the main screen is rather crowded [Figure 1], and moving the 3D model was sluggish (which could probably be attributed to my computer’s processor speed, which was below the minimum requirements as written on the box).

## FURTHER EXPLORATION

My initial enthusiasm decreased with prolonged use. The image quality itself remains very attractive and gives the application a high potential. There are numerous anatomical structures that can be selected and deselected, including surface overlays. All of these can be rotated by the user in all three dimensions. However, navigating the 3D brain is not always intuitive, and it may cause some effort to rotate the brain in the way you want to see it. The menu keeps distracting me, vessel names are projected over the brain in a way that limits readability [Figure 1], and the whole user interface makes my eyes jump across the screen – instead of quietly focussing on what it is all about.

## DISCUSSION

With a brand new, powerful computer that I bought a few months ago, I did not expect my hardware to be a limiting factor. To my surprise it was, which is both good and bad news. The good news is that over time, the general application’s performance will improve as hardware costs decrease. The bad news is that if you want to use the application today, you will need the most advanced – and therefore most expensive – hardware that is currently available. People who play a lot of video games or do graphic design work are likely to have such hardware, but this is not a typical profile for the application’s consumer market. Maybe at institutions, but not privately. So when considering the purchase of this application, first consider buying new hardware.

I would seriously advise the publisher to perform better usability testing among the target audience. Although I realize that this is a scientific program, it is also a final product and not a beta version. More attention to detail would be appreciated, or at least a decluttered user interface that allows full focus on the beautiful and informative graphical content. Lastly, at a retail price of approximately $190, I would have expected the developer to provide a custom application icon, instead of the default Adobe Director icon.

## CONCLUSION

The Cerefy® Atlas of Cerebral Vasculature is indeed “a state-of-the-art tool for visualizing and exploring the brain’s arterial and venous systems in three dimensions” as the box states. It is absolutely state-of-the-art when it comes to image quality. It is a little too much state-of-the-art when it comes to hardware requirements. But it is not state-of-the-art when it comes to usability, and therefore – unfortunately – the end-user experience. It is a high-potential application, but requires expensive hardware and needs better implementation to engage its users. An overview of the positive and negative aspects can be found in Table 1.

**Table 1:** Summary of positive and negative aspects

Positive	Negative
Superb image quality	Tough hardware requirements
Detailed anatomical structures	Cluttered user interface
Interactive anatomical subselections	3D navigation not very intuitive

